# Frailty and its influence on mortality and morbidity in COPD: A Systematic Review and Meta-Analysis

**DOI:** 10.1007/s11739-023-03405-6

**Published:** 2023-09-05

**Authors:** Alessia Verduri, Ben Carter, James Laraman, Ceara Rice, Enrico Clini, Nick Anthony Maskell, Jonathan Hewitt

**Affiliations:** 1 Department of Population Medicine, Cardiff University, Cardiff, UK; 2 Respiratory Unit, Department of Surgical and Medical Sciences, University of Modena and Reggio Emilia, Modena, Italy; 3 Department of Biostatistics and Health Informatics, Institute of Psychiatry, Psychology and Neuroscience, King’s College London, London, UK; 4 Academic Respiratory Unit, School of Clinical Sciences, University of Bristol, Bristol, UK

**Keywords:** COPD, Frailty, Mortality, Exacerbation, Hospitalization, Readmission

## Abstract

Frailty increases vulnerability to adverse outcomes. Long-term conditions increase the risk of frailty. We searched PubMed, Web of Science, The Cochrane Library, EMBASE from inception to March 2022. Quality assessment was conducted using the NOS. Data was analysed in a pooled a random-effects meta-analysis. Our primary outcome was the impact of frailty on mortality in adults with Chronic Obstructive Pulmonary Disease (COPD) diagnosis according to the guidelines. Secondary outcomes were: frailty and association with readmissions, hospitalisations, exacerbation rates, and prevalence of frailty in COPD. We identified 25 studies, with 5882 participants. The median prevalence of frailty was 47% (IQR, 39.3–66.3%, range 6.4–72%). There was an association between COPD patients living with frailty and increased risk of mortality versus COPD patients without frailty (pooled OR, 4.21 (95% CI 2.99–5.93,* I*^2^ 55%). A descriptive analysis of relationship between frailty and hospital readmission and all cause hospitalization showed positive associations. The relationship between frailty and the risk of exacerbation showed a pooled OR, 1.45 (95% CI 0.37–5.70, *I*^2^ 80%). Frailty is significantly associated with higher mortality risk in COPD. Frailty is common in patients with COPD and its measurement should be considered in clinical practice to better characterise COPD.

**Supplementary Information:**

The online version contains supplementary material available at 10.1007/s11739-023-03405-6.

## Introduction

Chronic Obstructive Pulmonary Disease (COPD) is one of the leading causes of mortality, morbidity, and health-care use worldwide (www.goldcopd.org). It is both preventable and treatable. COPD is one of the most common chronic diseases in old age. COPD diagnosis should be considered in any individual who has chronic dyspnea and cough and a history of exposure to risk factors such as smoking and must be functionally confirmed by spirometry. The disease is frequently associated with chronic comorbidities, including cardiovascular and metabolic disease, that may potentially influence health status and mortality of COPD patients. The prevalence of COPD increases with age and the highest rate is among those > 60 years. COPD prevalence data varies widely across countries which is likely due to different diagnostic criteria. The global prevalence of COPD according to the GOLD definition was 10.3% among people ages 30 to 79 years in 2019 [1]. The lowest estimates of prevalence are based on self-reported diagnosis, is under recognized and often misdiagnosed [2], these numbers underestimate the true prevalence. The burden of COPD is expected to increase over the next decades due to continued exposure to risk factors and ageing of the world’s population. The health care costs associated with COPD are high. In Europe, the direct costs of respiratory disease account for about 6% of health budgets and more than 50% of this is due to COPD. The disease contributes to significant health care burden annually in terms of visits, access to emergency departments and hospitalisations (www.goldcopd.org).

Frailty is a syndrome in which multiple factors reduce physiological capacity and increase an individual’s vulnerability to adverse health outcomes following minor stressor events [3, 4]. People living with frailty are at higher risk of falls, disability, prolonged hospitalisation, admission to care homes, and death [3, 4]. There are two main classifications of frailty (the deficit model and the phenotype model) and many clinical assessment tools used to measure it [4–6]. Some instruments use scoring systems and standardised cut-offs based on multiple domains including cognitive and social items, while others use a single functional measurement, such as hand grip strength [6–8]. Prevalence of frailty varies according to the criteria model used and the setting in which a population is studied [9].

Chronic diseases, such as lung diseases, are known risk factors for the development of frailty [6, 9]. Not surprisingly frailty is common in people with COPD. The prevalence of frailty in the COPD population varied from 9 to 64% according to the criteria of the phenotype model and from 9 to 28% in studies based on different frailty models (Marengoni et al*.*) [10]. The literature on frailty in COPD is evolving and frailty in COPD is common in older patients both in primary and secondary care settings [11]. The systematic review and meta-analysis on the relationship between COPD and frailty, published by Marengoni et al. in 2018 [10], demonstrated a two-fold increase of being frail if one has COPD, compared to people without COPD. These data did not explore a link between adverse outcomes associated with frailty and people living with COPD.

The identification of outcomes in patients with COPD living with frailty is important to predict disease progression and improve clinical outcomes [12, 13]. The primary outcomes of this review were to assess the prevalence of frailty in a population with COPD and to determine the association between frailty and mortality in people with COPD. The secondary outcomes were to explore the association between frailty and readmissions, hospitalisations, and exacerbations.

## Study design and methods

The systematic review and meta-analysis were conducted in accordance with the Preferred Reporting Items for Systematic Reviews and Metanalyses (PRISMA) recommendations. The protocol was registered through the PROSPERO database (registration number: CRD42022328511).

### Search strategy

The search strategy was developed in partnership with a specialist librarian. Two researchers (AV, JL) independently searched four electronic databases (PubMed, Web of Science, The Cochrane Library and EMBASE) for manuscripts published from inception to 24th March 2022. The search terms were based on Medical Subject Headings (MeSH) and the following words referring to frailty and COPD were used as keywords: Pulmonary Disease, Chronic Obstructive Bronchitis, Emphysema, AND frailty. The search strategy is outlined in Supplementary Information (SI) file. Studies reporting information on frailty assessment and COPD in titles and abstracts were included. Any disagreement on study eligibility was resolved through discussion with a third reviewer (JH). A hand search of the reference lists of all relevant articles was performed to identify any articles not captured by the electronic search. Only full-text reports were considered reported in English.

**Fig. 1:**
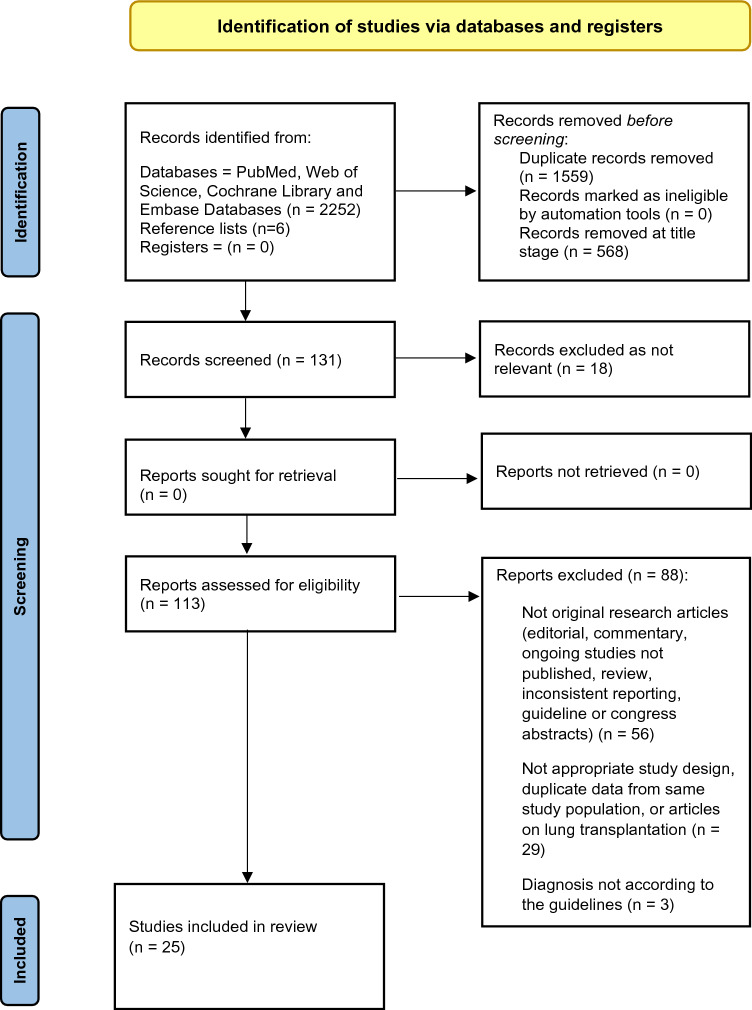
PRISMA Flowchart of the included studies on COPD. *From:* Page MJ, McKenzie JE, Bossuyt PM, Boutron I, Hoffmann TC, Mulrow CD, et al (2021) The PRISMA 2020 statement: an updated guideline for reporting systematic reviews. BMJ 372:n71

The inclusion criteria were: (1) Study participants (≥ 18 years) who were diagnosed with COPD and were assessed for frailty. We accepted a diagnosis of COPD according to the recognised international GOLD guidelines (www.goldcopd.org) confirmed by spirometry (post-bronchodilator FEV_1_/FVC < 0.70). Studies with confirmed diagnosis of COPD according to the International Classification of Diseases (ICD-Codes; www.who.int/standards/classifications/classification-of-diseases) were also included; (2) Only studies using a validated method of frailty identification were included; (3) Outcome measures were: prevalence of frailty and related- mortality and morbidity in patients with COPD; (4) Cross-sectional, longitudinal, prospective or retrospective cohort and case–control study designs. The review process is summarised in a PRISMA flow diagram (Fig. 1). Exclusion criteria were patients with COPD listed for lung transplantation.

### Data extraction and quality assessment

Study characteristics, demographic information, frailty tool, frailty prevalence and outcomes data were independently extracted from the included studies. Study authors were contacted to clarify or provide additional data where it was missing or unclear.

For the studies included the quality assessment was conducted by the two reviewers independently (AV, JL) and arbitrated by a third (JH) using the Newcastle–Ottawa Scale (NOS) [14], which assesses the risk of bias in observational studies. Each domain examined was classified as good, fair or poor. Studies were considered to be of good quality where they scored good for all domains, fair if they scored fair in one or more domain and poor if they scored poorly in any domain.

### Outcomes

The primary outcome was mortality in people with COPD and frailty. A priori, *long-term mortality* was defined as ≥ 1-year mortality, and *short-term mortality* was defined as in-hospital mortality or 30- or 90-day mortality after admission for acute exacerbation of COPD. Secondary outcomes were: readmissions to hospital within 30 days or 90 days following hospitalisation for AECOPD; hospitalisations due to any-cause or COPD; and COPD exacerbations rates (patients experiencing at least one COPD exacerbation).

### Key exposure of frailty

Studies were included if they measured frailty using any validated instrument (this could be either a deficit index or clinical judgement approach). The following were examples of those included: Fried Frailty Phenotype [4], Frailty Index [5], Clinical Frailty Scale (CFS) [8], FRAIL Scale [15], Canadian Study of Health and Aging Clinical Frailty Scale [16], Reported Edmonton Frailty Scale [17], Kihon Checklist [18], FiND questionnaire [19], PRISMA-7 questionnaire [20], Tilburg Frailty Indicator [21], and Frailty Staging System [22]. Frailty was measured as a binary variable as frail or not frail using the thresholds presented for the individual instruments. For example, within the CFS 4–9 were scored as frail compared to 1–3 that were not frail. Studies using Gait Speed as a surrogate indicator of physical frailty and Time Up and Go Test to assess risk of falls were excluded. We summarised each of frailty instruments in SI-Table 1D.

**Table 1:** Included studies on COPD

Author	Year	Country	Population details	No. patients	Age*	Sex (% and No. M and F)	COPD diagnosis/definition criteria	Frailty measure	Quality assessment	NOS
*Studies on COPD and frailty*										
Oishi et al	2020	Japan	COPD outpatients in stable condition (cross-sectional study)	128	≥ 40 yrs 73 (69–78)	91.4% 117 M 8.6% 11 F	GOLD guidelines	Kihon checklist	Fair	5
Takahashi et al	2021	Japan	COPD outpatients in stable condition (cross-sectional study)	40	No age cut off 70.6 ± 8.2	97.5% 39 M 2.5% 1 F	GOLD guidelines	Kihon checklist	Fair	5
Nishimura et al	2021	Japan	COPD outpatients in stable condition (cross-sectional study)	89	≥ 50 yrs 78 (74–82)	93.2% 83 M 6.8% 6 F	GOLD guidelines	Kihon checklist	Fair	5
Kagiali et al	2021	Turkey	COPD outpatients in stable condition (cross-sectional study)	48	≥ 55 yrs 67.3 ± 5.1 Frail COPD patients 65.1 ± 4.6 Non-frail COPD patients	80% 38 M 20% 10 F	Post-bronchodilator FEV_1_/FVC 0.7	Fried frailty phenotype	Fair	6
Dias et al	2020	Brazil	COPD outpatients in stable condition (cross-sectional study)	153	≥ 40 yrs 68.8 (60.5–80.5)	55% 84 M 45% 69 F	GOLD guidelines	FRAIL scale	Good	7
Gale et al	2018	UK	COPD outpatients in stable condition (cross-sectional study)	520 COPD 150 controls	No age cut off 66.1 ± 7.6	52% 270 M 48% 250 F	GOLD guidelines	Frailty Index-comprehensive geriatric assessment	Good	7
Medina-Mirapeix et al	2018	Spain	COPD outpatients in stable condition (cross-sectional study)	137	Range 40–80 66.9 ± 8.3	87.6% 120 M 12.4% 17 F	GOLD guidelines	Fried frailty phenotype	Fair	6
Naval et al	2021	Spain	COPD outpatients in stable condition (cross-sectional study)	127	≥ 40 yrs 66.5 ± 7.9	85% 108 M 15% 19 F	GOLD guidelines	Fried frailty phenotype	Fair	6
Hirai et al	2019	Japan	COPD outpatients in stable condition (cross-sectional study)	201	≥ 65 yrs 76 (70–81)	87% 175 M 13% 26 F	GOLD guidelines	Kihon checklist	Fair	5
Chen et al	2018	Taiwan	COPD outpatients divided into dyspnea and non-dyspnea group (cross-sectional study)	125	No age cut off 76.3 ± 10.2	100% 125 M	GOLD guidelines	Canadian study of health and aging clinical frailty scale (modified score as a binomial variable: ≤ 3 non frail and ≥ 4 frail)	Fair	4
Mustafaoğlu et al	2020	Turkey	COPD outpatients in stable condition (cross-sectional study)	61	Range 65–84	97% 59 M 3% 2 F	GOLD guidelines	Tilburg frailty indicator	Fair	5
ter Beek et al	2020	The Netherlands	COPD outpatients starting pulmonary rehabilitation (cross-sectional study)	57	≥ 40 yrs 61.2 ± 8.7	49% 28 M 51% 29 F	GOLD guidelines	Fried frailty phenotype	Fair	5
Finamore et al	2021	Italy	COPD outpatients during and after pulmonary rehabilitation (prospective study)	53	No age cut off 73 ± 8	49% 26 M 51% 27 F	GOLD guidelines (grades 1–3)	PRISMA-7 questionnaire	Fair	6
Gephine et al	2021	Canada	COPD outpatients with chronic respiratory failure in stable condition, starting pulmonary rehabilitation (prospective study)	44	≥ 40 yrs 66 ± 8	68% 30 M 32% 14 F	GOLD guidelines	Fried frailty phenotype	Fair	6
*Cohort studies on COPD outpatients and frailty*										
Yee et al	2020	US	COPD outpatients in stable condition (prospective study)	280	≥ 40 yrs mean age 68	80% 224 M 20% 56 F	Post-bronchodilator FEV_1_/FVC 0.7 and FEV_1_ < 80% predicted	Fried frailty phenotype	Good	9
Scarlata et al	2021	Italy	COPD outpatients in stable condition (retrospective study)	150	≥ 60 yrs 73 ± 8	72% 107 M 28% 43 F	GOLD guidelines	Frailty index	Good	9
Luo et al	2021	China	COPD outpatients in stable condition (prospective study)	309	≥ 65 yrs 86 (80–90)	78% 241 M 22% 68 F	GOLD guidelines	Fried frailty phenotype	Good	9
Kennedy et al	2019	US	Analysis on 2-year survival from randomized control trial -NETT study (retrospective study)	902	67 (63–70)	62.4% 563 M 37.6% 339 F	COPD patients with severe emphysema	Fried frailty phenotype	Good	9
*Cohort studies on patients admitted with acute exacerbation of COPD*										
Warwick et al	2021	Canada	COPD patients admitted to ICU for acute exacerbation (retrospective study)	390	≥ 18 yrs 68.2 ± 11.1	47.2% 184 M 52.8% 206 F	Classification of Diseases 10^th^ Edition codes (ICD-10)	Clinical frailty scale	Good	8
Gu et al	2021	China	COPD patients admitted for acute exacerbation (retrospective study)	154	≥ 60 yrs 79.7 ± 8.3	71% 109 M 29% 45 F	Post-bronchodilator FEV_1_/FVC 0.7 and FEV_1_ < 80% predicted	Frailty index-lab	Fair	6
Alqahtani et al	2021	UK	COPD patients admitted for acute exacerbation (prospective study)	82	No age cut off 71 ± 10.4	49% 40 M 51% 42 F	Post-bronchodilator FEV_1_/FVC 0.7	Reported edmonton frail scale	Good	9
Witt et al	2021	US	COPD patients admitted for acute exacerbation (prospective study)	70	≥ 18 yrs 63.5 (58–71)	44% 31 M 56% 39 F	FEV_1_ and FVC by spirometry, lung obstruction (not mentioned bronchodilation)	Fried frailty phenotype	Good	9
*Studies on community-dwelling adults and frailty*										
Castellana et al	2021	Italy	Community-dwelling adults (cross-sectional and longitudinal study)	1929 343 COPD	≥ 65 yrs 73.5 ± 6.2	50.4% 974 M 49.6% 955 F	Post-bronchodilator FEV_1_/FVC 0.7, COPD prevalence 17.8%	Fried frailty phenotype	Fair	5
Lee et al	2021	Singapore	Community-dwelling adults (prospective cohort study)	4627 1162 COPD	≥ 55 yrs 66.4 ± 7.7	37% 1711 M 63% 2916 F	GOLD guidelines, COPD prevalence 25.1%	Fried frailty phenotype	Good	9
Ierodiakonou et al	2019	Greece	Primary care (cross-sectional study)	257	No age cut off 65 ± 12.3	79.4% 204 M 20.6% 53 F	GOLD guidelines	FiND questionnaire	Good	7

*Expressed by Mean ± Standard Deviation (SD) or Median (IQR)

### Data analysis

The prevalence of frailty was estimated as the median study level prevalence, presented alongside the interquartile range.

The primary outcome of mortality was associated with frailty. Only studies that were clinically and contextually homogenous were considered for pooling. Homogenous studies were pooled using a Mantel–Haenszel method with a random-effects. Pooled effects were presented as odds ratio (OR) with associated 95% CIs, *p*-values, and *I*^2^ summary data. All pooled meta-analyses were performed using Review Manager Version 5.4.

Secondary outcomes were narratively described and, where study characteristics were deemed as contextually homogeneous, they were associated with frailty as a binary variable. Where possible secondary outcomes were pooled in a manner consistent with the primary outcome.

### Assessment of subgroups and statistical heterogeneity

Statistical heterogeneity was measured using the *I*^2^ statistic. Heterogeneity exceeding 80% was explored using subgroup analyses. Pre-specified subgroups to explore heterogeneity included; age; gender; study design, type of frailty instrument, and study level risk of bias.

## Results

### Search results and quality assessment

After removal of duplicates, 699 records were identified. One hundred and thirteen (113) full texts were reviewed, and 88 of these were excluded for the following reasons: not original research articles (editorial, commentary, ongoing studies not published, review, inconsistent reporting, guideline or congress abstracts); not appropriate study design; duplicate data from same study population; articles on lung transplantation; diagnosis not according to the guidelines. Twenty-five (25) studies included are shown in the PRISMA flowchart (Fig. 1). Eleven studies were determined as good quality [23–33], fourteen were categorised as fair quality [34–47]. For further details of the quality assessments tool, see SI-Table 1A, SI- Table 1B, and SI- Table 1C.

### Characteristics of the included studies

The included studies were published between 2018 and 2022, and of the 25 studies, 11 were cohort studies and 14 were cross-sectional (Table 1). The studies originated from Japan [34–36, 40], Taiwan [41], China [28, 45], Singapore [32], Turkey [47], Greece [33], Italy [26, 43, 46], Spain [38, 39], the United Kingdom [24, 30], the Netherlands [42], Brazil [23], USA [25, 29, 31], and Canada [27, 44]. Of the 25 studies, 5882 participants were included, 46.5% were male (2735/5882). There was a range of frailty assessment tools used in the included studies, of which 22 were deemed suitable for inclusion in the frailty prevalence estimation. The average age of the participants was 70 years old; this does not include the study by Mustafaoğlu et al*.* [47] as the mean age was not recorded, although the study reported age range 65–84 (Table 1).

### Frailty prevalence in COPD

Prevalence was assessed using 11 different frailty scales, with the most common being the Fried Frailty Phenotype, the Frailty Index, and the Kihon Checklist. Two of the 25 included studies [30, 41] did not report data on prevalence of frailty in COPD. Of 29 included studies, the median prevalence of frailty in COPD patients was 47 (IQR, 39.3–66.3; range 6.4–72%) (SI- Table 1D and SI- Table 1E). The overall prevalence of pre-frailty ranged between 19.6 and 73.7%. Only one study, Gephine et al*.* [44], included patients with COPD with chronic respiratory failure defined as use of long-term oxygen therapy and/or non-invasive ventilation. The overall prevalence of frailty in COPD was 43% (SI- Table 1C).

### Mortality

Seven longitudinal studies explored the relationship between frailty and *long-term* mortality across 2560 participants [51]. Five studies [28, 29, 32, 50, 52] found a positive association, whilst two studies [25, 26] reported no association.

Two studies [27, 45] explored the influence of frailty on *short-term* mortality across 544 patients. Both studies reported an association between being frail and mortality.

**Fig. 2:**
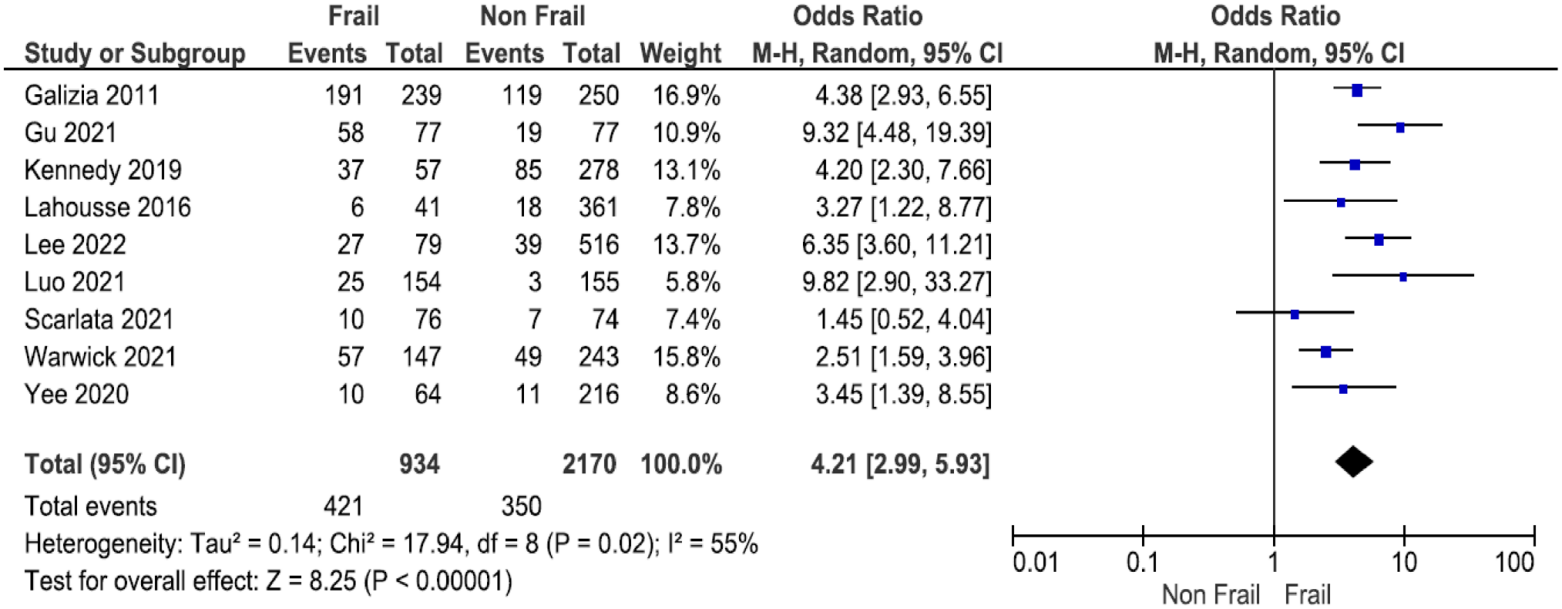
A meta-analysis of frailty and mortality in COPD. Forest plot demonstrating effect of frailty on all-cause short- and long-term mortality. The mortality of patients with COPD living with or without frailty were compared across nine studies

Overall, there was an association found with patients with COPD living with frailty and increased risk of mortality compared to patients with COPD without frailty pooled OR, 4.21 (95% CI 2.99–5.93,* I*^2^ 55%) (Fig. 2).

One study, Kennedy et al*.* [29], conducted a retrospective analysis of 2-year survival data from the 5-year, multicentre study. They found a significantly reduced survival in frail participants with a mortality rate of 36% compared to 16% in non-frail or pre-frail participants. Therefore, we performed a subgroup analysis of the six studies which reported long-term mortality only [25, 26, 28, 32, 50, 52], excluding the data of the Kennedy study [29]. The results showed OR, 0.16 (95% CI 0.07–0.25,* I*^2^ 84%) (SI-Fig. 3).

### Readmission

Three studies [30, 31, 48] investigated readmissions within 30 and 90 days. Of three studies involving 255 patients, all of them [30, 31, 48] found positive associations between frailty and readmission. Witt et al*.* [31] demonstrated an Odds Ratio of 19.31 (1.07–349.03) in a small sample (*n* = 70) of which all 8 readmitted patients were living with frailty. Alqahtani and colleagues [30] demonstrated that people who were readmitted had a higher frailty index than those who were not admitted but did not provide data on the exact numbers of people readmitted. Bernabeu-Mora and colleagues [48] reported a trend (*p* = 0.002) showing increased readmission with increasing frailty [49].

### Hospitalisation

Three studies [25, 28, 29] investigated hospitalisations, which included 1491 participants in stable condition. All of these studies reported at some evidence of an association between frailty and hospitalisation. Yee and colleagues [25] demonstrated that frailty phenotype was associated only with non-COPD-related hospitalisations (Incidence Rate Ratio 2.62, 95%CI 1.00–6.84, *p* = 0.05). Luo et al*.* [28] showed that all-cause hospitalisations were significantly higher in the frail group versus the non-frail group (*p* < 0.001). The study of Kennedy et al. [29] found that frailty phenotype was associated with increased incidence of hospitalisations (*p* = 0.02).

### Exacerbations

Three studies [25, 32] explored the relationship between frailty and risk of *future* moderate-to-severe exacerbations within the next year. Two studies, including 1751 patients, showed no association. One study [28] found a positive association (*n* = 309) showing that the risk of moderate-to-severe acute exacerbation within one year was higher in patients with COPD and frailty compared to patients without frailty (*p* < 0.001).

Of the included studies, eight [23, 24, 26, 28, 33, 38, 44] across 1697 participants investigated the relationship between frailty and the number of COPD exacerbations in the *past* year. Only three studies [28, 38, 44] reported the exact number of patients with ≥ 1 or ≥ 2 exacerbations of COPD within the last year. Only one [28] of these three studies found that, in 309 patients with COPD, people living with frailty had more previous exacerbations than those living without frailty (*p* < 0.001). Overall, there was no association found with patients with COPD living with frailty and increased risk of previous exacerbations compared to patients with COPD without frailty (pooled OR, 1.45 95%CI 0.37–5.70, *I*^2^ 80%) (SI-Fig. 4).

## Discussion

The main objective of this review was mortality in people with COPD and frailty. The secondary aims were prevalence of frailty in COPD and influence of frailty on hospitalisations, readmissions, and exacerbations in patients with COPD. This study identified 25 studies with 5882 patients. Eleven studies were good quality, and the remaining fourteen fair quality.

This is the first study to systematically review the literature on mortality and frailty in patients with COPD. We demonstrated that all-cause mortality in COPD was associated with being frail in both the short and longer term. Most existing evidence on long-term mortality in COPD includes studies that assess patients hospitalised for AECOPD, both in general medicine wards and Intensive Care Units [53–56]. The systematic review of Singanayagam et al*.* [57] concluded that short-term mortality, including in-hospital mortality, was influenced by multiple factors in hospitalised patients for AECOPD such as age and comorbidities which could be inferred as similar to the syndrome of frailty.

The study demonstrated that nearly 50% of patients with COPD, diagnosed using the GOLD criteria, were living with frailty. The frailty instruments used in these studies (Fried model and Frailty Index) [4, 5] are have been extensively validated in people aged over 65 years [58]. In younger populations, they have been used although much less widely [59], therefore generalizing to younger populations with COPD, should be done with caution.

For the prevalence estimate, we included three studies [42–44] that explored the relationship between frailty and pulmonary rehabilitation in patients with COPD. Finamore et al*.* [43] confirmed the influence of frailty on the walking distance during and after the programme and showed greater improvement in rehabilitation outcomes in frail patients compared to non-frail patients [60]. These findings are in line with the study of Maddocks et al*.* [12]. At the start of rehabilitation programme, ter Beek et al*.* [42] found high coexistence of malnutrition and frailty in participants with COPD. Gephine et al*.* [44] reported a greater use of nutritional supplements in patients with COPD with chronic respiratory failure and frailty. Nutritional status is one of the components of assessment in pulmonary rehabilitation and is important in determining frailty. Rehabilitation programmes that improve levels of physical activity and malnutrition can increase quality of life and reduce number of hospitalisations and mortality in patients with COPD [www.goldcopd.org, 60]. These programmes should be recommended to make lifestyle changes that might potentially decrease or reverse frailty in COPD. While not the focus of this systematic review, these studies highlight the interventions to ameliorate the high prevalence of frailty in this population can be beneficial. This emphasizes the need for pulmonary rehabilitation and frailty be studied further in people living with COPD.

The study showed there is some evidence of positive association between the frailty phenotype and hospitalization in patients with COPD. Also, the review found that readmissions to hospital within three months after acute exacerbation were more frequent in patients with COPD and frailty. Although these findings require future studies and larger samples to explore better these relationships, these results are consistent with frailty adversely contributes to a range of poor outcomes in COPD, which would be in line with the literature.

The study investigated the role of frailty and the risk of exacerbations of COPD, with no convincing associations demonstrated. However, only a few studies were identified, confirming the need for further longitudinal reports in this population. Specifically, only a small number of studies reported the exact number of patients who had an exacerbation and the exact number of those exacerbations. Most authors reported the mean or median value of exacerbations making meta-analyses difficult and we would urge future authors to report these detailed data.

A strength of this review is the use of GOLD guidelines for the diagnosis of COPD. These guidelines are the internationally recommended standard for the diagnosis and management of COPD. COPD is commonly self-reported, which is known to underestimate the true prevalence of disease. Therefore, this review provides by far the most accurate estimate of frailty with COPD. This systematic review had some limitations. First, the review included only nonrandomized studies and reverse causality cannot be ignored. Also, while we only considered recognised frailty tools for the diagnosis of frailty, we considered 11 different frailty instruments. Hence some heterogeneity cannot be excluded. However, whilst the different frailty tools may have offered the contextual diversity, they were not able to explain the heterogeneity. This represents an additional weakness of the review. In addition, the length of follow up in the included studies may have explained some of the heterogeneity found in the analysis of long-term mortality.

Future research should explore frailty as a modifiable risk factor and the development of clinical interventions to reverse the effect of frailty such as pulmonary rehabilitation that may, potentially, reduce health-care use and rate of admissions in routine practice.

## Conclusion

The study shows a high prevalence of frailty in people with COPD diagnosed according to GOLD criteria. Our review suggests that frailty has a clear association with mortality in COPD. This data can be used to support shared decision-making in hospital settings. Our findings highlight the need of early identification of patients with COPD living with frailty to minimise their risk. Further work is urgently needed to identify a single frailty assessment tool that includes physical, cognitive and social domains for patients with COPD to accurately capture the complexity of the condition.

### Supplementary Information

Below is the link to the electronic supplementary material.

Supplementary file1 (DOC 350 KB)

## Data Availability

All data sharing and collaboration requests should be directed to the corresponding author. The data underlying this article are available in the article and in its online Supplementary Information file.

## Declarations

### Research in context

Evidence before this study: Frailty is common in people with COPD. A previous systematic review on the relationship between COPD and frailty demonstrated a two-fold increase of being frail if one has COPD, compared to people without COPD. These data did not explore a link between adverse outcomes associated with frailty and people living with COPD. Added value of this study: This is the first study to systematically review the literature on mortality and frailty in patients with confirmed diagnosis of COPD. These data demonstrated that all-cause mortality in COPD is associated with being frail in both the short and longer term. Implications of all the available evidence: All-cause mortality in COPD is associated with being frail in both the short and longer term. Frailty assessment should be considered in routine daily practice in all people living with COPD.

## References

[1] Adeloye D, Song P, Zhu Y (2022). Global, regional, and national prevalence of, and risk factors for, chronic obstructive pulmonary disease (COPD) in 2019: a systematic review and modelling analysis. Lancet Respir Med.

[2] Verduri A, Hewitt J, Carter B, et al (2022) Prevalence of asthma and COPD in a cohort of patients at the follow up after COVID-19 pneumonia. Pulmonology 10:S2531–0437(22)00127–1 10.1016/j.pulmoe.2022.05.005 PMC9186410 35798643

[3] Clegg A, Young J, Iliffe S (2013). Frailty in elderly people. Lancet.

[4] Fried LP, Tangen CM, Walston J (2001). Frailty in older adults: evidence for a phenotype. J Gerontol A Biol Sci Med Sci.

[5] Mitnitski AB, Mogilner AJ, Rockwood K (2001). Accumulation of deficits as a proxy measure of aging. Sci World J.

[6] Morley JE, Vellas B, van Kan GA (2013). Frailty consensus: a call to action. J Am Med Dir Assoc.

[7] Aguayo GA, Donneau AF, Vaillant MT (2017). Agreement between 35 published frailty scores in the general population. Am J Epidemiol.

[8] Rockwood K, Song X, MacKnight C (2005). A global clinical measure of fitness and frailty in elderly people. CMAJ.

[9] Buta BJ, Walston JD, Godino JG (2016). Frailty assessment instruments: systematic characterization of the uses and contexts of highly-cited instruments. Ageing Res Rev.

[10] Marengoni A, Vetrano DL, Manes-Gravina E (2018). The relationship between COPD and frailty: a systematic review and meta-analysis of observational studies. Chest.

[11] Mirza S, Benzo R (2017). Chronic obstructive pulmonary disease phenotypes: implications for care. Mayo Clin Proc.

[12] Maddocks M, Kon SS, Canavan JL (2016). Physical frailty and pulmonary rehabilitation in COPD: a prospective cohort study. Thorax.

[13] Bone AE, Hepgul N, Kon S (2017). Sarcopenia and frailty in chronic respiratory disease. Chron Respir Dis.

[14] Stang A (2010). Critical evaluation of the Newcastle-Ottawa scale for the assessment of the quality of nonrandomized studies in meta-analyses. Eur J Epidemiol.

[15] Abellan van Kan G, Rolland YM, Morley JE (2008). Frailty: toward a clinical definition. J Am Med Dir Assoc.

[16] Chan DC, Tsou HH, Chen CY (2010). Validation of the Chinese-Canadian study of health and aging clinical frailty scale (CSHA-CFS) telephone version. Arch Gerontol Geriatr.

[17] Rolfson DB, Majumdar SR, Tsuyuki RT (2006). Validity and reliability of the Edmonton frail scale. Age Ageing.

[18] Ogawa K, Fujiwara Y, Yoshida H (2011). The validity of the "Kihon Check-list" as an index of frailty and its biomarkers and inflammatory markers in elderly people. Nihon Ronen Igakkai Zasshi.

[19] Cesari M, Demougeot L, Boccalon H (2014). A self-reported screening tool for detecting community-dwelling older persons with frailty syndrome in the absence of mobility disability: the FiND questionnaire. PLoS One.

[20] Raiche M, Hebert R, Dubois MF (2008). PRISMA-7: a case-finding tool to identify older adults with moderate to severe disabilities. Arch Gerontol Geriatr.

[21] Gobbens RJ, van Assen MA, Luijkx KG (2010). The Tilburg Frailty Indicator: psychometric properties. J Am Med Dir Assoc.

[22] Cacciatore F, Abete P, Mazzella F (2005). Frailty predicts long-term mortality in elderly subjects with chronic heart failure. Eur J Clin Invest.

[23] Dias LS, Ferreira ACG, da Silva Junior JLR (2020). Prevalence of frailty and evaluation of associated variables among COPD patients. Int J Chron Obstruct Pulmon Dis.

[24] Gale NS, Albarrati AM, Munnery MM (2018). Frailty: A global measure of the multisystem impact of COPD. Chron Respir Dis.

[25] Yee N, Locke ER, Pike KC (2020). Frailty in chronic obstructive pulmonary disease and risk of exacerbations and hospitalizations. Int J Chron Obstruct Pulmon Dis.

[26] Scarlata S, Finamore P, Laudisio A (2021). Association between frailty index, lung function, and major clinical determinants in chronic obstructive pulmonary disease. Aging Clin Exp Res.

[27] Warwick M, Fernando SM, Aaron SD (2021). Outcomes and resource utilization among patients admitted to the intensive care unit following acute exacerbation of chronic obstructive pulmonary disease. J Intensive Care Med.

[28] Luo J, Zhang D, Tang W (2021). Impact of frailty on the risk of exacerbations and all-cause mortality in elderly patients with stable chronic obstructive pulmonary disease. Clin Interv Aging.

[29] Kennedy CC, Novotny PJ, LeBrasseur NK (2019). Frailty and clinical outcomes in chronic obstructive pulmonary disease. Ann Am Thorac Soc.

[30] Alqahtani JS, Aldabayan YS, Aldhahir AM (2021). Predictors of 30- and 90-Day COPD exacerbation readmission: a prospective Cohort study. Int J Chron Obstruct Pulmon Dis.

[31] Witt LJ, Spacht WA, Carey KA (2021). Weak handgrip at index admission for acute exacerbation of COPD predicts all-cause 30-day readmission. Front Med (Lausanne).

[32] Lee SY, Nyunt MSZ, Gao Q (2022). Co-occurrence of physical frailty and COPD and association with disability and mortality: Singapore longitudinal ageing study. Chest.

[33] Ierodiakonou D, Kampouraki M, Poulonirakis I (2019). Determinants of frailty in primary care patients with COPD: the Greek UNLOCK study. BMC Pulm Med.

[34] Oishi K, Matsunaga K, Harada M (2020). A new dyspnea evaluation system focusing on patients’ perceptions of dyspnea and their living disabilities: The linkage between COPD and Frailty. J Clin Med.

[35] Takahashi S, Hirano T, Yasuda K (2021). Impact of frailty on hippocampal volume in patients with chronic obstructive pulmonary disease. Biomedicines.

[36] Nishimura K, Nakayasu K, Mori M (2021). Are fatigue and pain overlooked in subjects with stable chronic obstructive pulmonary disease?. Diagnostics (Basel).

[37] Kagiali S, Inal-Ince D, Cakmak A (2022). Daily living activities, exercise capacity, cognition, and balance in COPD patients with and without frailty. Ir J Med Sci.

[38] Medina-Mirapeix F, Bernabeu-Mora R, Gimenez-Gimenez LM (2018). Physical frailty characteristics have a differential impact on symptoms as measured by the CAT score: an observational study. Health Qual Life Outcomes.

[39] Naval E, Gonzalez MC, Giraldos S (2021). Frailty assessment in a stable COPD cohort: is there a COPD-frail phenotype?. COPD.

[40] Hirai K, Tanaka A, Homma T (2019). Comparison of three frailty models and a sarcopenia model in elderly patients with chronic obstructive pulmonary disease. Geriatr Gerontol Int.

[41] Chen PJ, Yang KY, Perng WC (2018). Effect of dyspnea on frailty stages and related factors in Taiwanese men with COPD. Int J Chron Obstruct Pulmon Dis.

[42] ter Beek L, van der Vaart H, Wempe JB (2020). Coexistence of malnutrition, frailty, physical frailty and disability in patients with COPD starting a pulmonary rehabilitation program. Clin Nutr.

[43] Finamore P, Scarlata S, Delussu AS (2021). Frailty impact during and after pulmonary rehabilitation. COPD.

[44] Gephine S, Mucci P, Grosbois JM (2021). Physical frailty in COPD Patients with chronic respiratory failure. Int J Chron Obstruct Pulmon Dis.

[45] Gu JJ, Liu Q, Zheng LJ (2021). A frailty assessment tool to predict in-hospital mortality in patients with acute exacerbations of chronic obstructive pulmonary disease. Int J Chron Obstruct Pulmon Dis.

[46] Castellana F, Lampignano L, Bortone I (2020). Physical frailty, multimorbidity, and all-cause mortality in an older population from southern italy: results from the Salus in Apulia Study. J Am Med Dir Assoc.

[47] Mustafaoğlu BT, Gulen ST, Birtekokak F (2020). Factors affecting frailty syndrome in elderly chronic obstructive pulmonaary disease patients and its relationship with systemic inflammation. Turk Geriatri Dergisi.

[48] Bernabeu-Mora R, Garcia-Guillamon G, Valera-Novella E (2017). Frailty is a predictive factor of readmission within 90 days of hospitalization for acute exacerbations of chronic obstructive pulmonary disease: a longitudinal study. Ther Adv Respir Dis.

[49] Kusunose M, Oga T, Nakamura S (2017). Frailty and patient-reported outcomes in subjects with chronic obstructive pulmonary disease: are they independent entities?. BMJ Open Resp Res.

[50] Galizia G, Cacciatore F, Testa G (2011). Role of clinical frailty on long-term mortality of elderly subjects with and without chronic obstructive pulmonary disease. Aging Clin Exp Res.

[51] Limpawattana P, Putraveephong S, Inthasuwan P (2017). Frailty syndrome in ambulatory patients with COPD. Int J Chron Obstruct Pulmon Dis.

[52] Lahousse L, Ziere G, Verlinden VJA (2016). Risk of frailty in elderly with COPD: a population-based study. J Gerontol A Biol Sci Med Sci.

[53] Suissa S, Dell’Aniello S, Ernst P (2012). Long-term natural history of chronic obstructive pulmonary disease: severe exacerbations and mortality. Thorax.

[54] Garcia-Sanz MT, Canive-Gomez JC, Senin-Rial L (2017). One-year and long-term mortality in patients hospitalized for chronic obstructive pulmonary disease. J Thorac Dis.

[55] Gudmundsson G, Ulrik CS, Gislason T (2012). Long-term survival in patients hospitalized for chronic obstructive pulmonary disease: a prospective observational study in the Nordic countries. Int J Chron Obstruct Pulmon Dis.

[56] Owusuaa C, Dijkland SA, Nieboer D (2022). Predictors of mortality in chronic obstructive pulmonary disease: a systematic review and meta-analysis. BMC Pulm Med.

[57] Singanayagam S, Schembri S, Chalmers JD (2013). Predictors of mortality in hospitalized adults with acute exacerbation of chronic obstructive pulmonary disease. Ann Am Thorac Soc.

[58] Hewitt J, Carter B, Vilches-Moraga A (2020). The effect of frailty on survival in patients with COVID-19 (COPE): a multicentre, European, observational cohort study. Lancet Public Health.

[59] Hanlon P, Lewsey J, Quint JK (2022). Frailty in COPD: an analysis of prevalence and clinical impact using UK Biobank. BMJ Open Respir Res.

[60] Garcia-Aymerich J, Lange P, Benet M (2006). Regular physical activity reduces hospital admission and mortality in chronic obstructive pulmonary disease: a population based cohort study. Thorax.

