# Supplementary material for: Frailty and its influence on mortality and morbidity in COPD: A Systematic Review and Meta-Analysis
Source: Intern Emerg Med. 2023 Sep 5;18(8):2423–34. doi: 10.1007/s11739-023-03405-6 (PMC10635928; doi:10.1007/s11739-023-03405-6)
Supplement: Supplementary file 1 — Supplementary file1 (DOC 350 KB) [file 11739_2023_3405_MOESM1_ESM.doc]

**Supplementary Information (SI)**

**Frailty and its influence on mortality and morbidity in COPD: A systematic review and Meta-Analysis.**

**Internal and Emergency Medicine**

Alessia Verduri,^1,2^ Ben Carter,^3^ James Laraman,^1^ Ceara Rice,^1^ Enrico Clini,^2^ Nick A. Maskell^4^ and Jonathan Hewitt^1^

**Corresponding author:**

Dr Alessia Verduri

Division of Population Medicine, Cardiff University, Cardiff (UK) & Respiratory Unit, Department of Surgical and Medical Sciences, University of Modena and Reggio Emilia, Modena (Italy) - [alessia.verduri@unimore.it](mailto:alessia.verduri@unimore.it)

**SI-Table 1A. Newcastle-Ottawa Score for the included studies on COPD.**

**SI-Table 1B. Newcastle-Ottawa Score (NOS) domain description.**

**SI-Table 1C**. **The Included Studies on COPD for Frailty Prevalence Estimation.**

**SI-Table 1D**. **The frailty assessment tools used in the included studies**.

**SI-Table 1E**. **Studies included in Marengoni *et al.* (2018) and considered in the analysis of frailty prevalence estimate, mortality and readmission.**

**SI-Figure 3. Subgroup sensitivity analysis of frailty on all-cause long-term mortality in COPD across six studies.**

**SI-Figure 4. A meta-analysis of frailty and past exacerbations in COPD across three studies.**

**SI-Table 1F. Search strategy.**

**SI-Table 1G. PRISMA 2020 Checklist.**

**SI-Table 1A. Newcastle-Ottawa Score for the included studies on COPD.**

|  | Selection^&^ | | | | Comparability* | | Outcome* | | | Quality assessment** | Notes |
| --- | --- | --- | --- | --- | --- | --- | --- | --- | --- | --- | --- |
|  | **1** | **2** | **3** | **4** | **1** | **2** | **1** | **2** | **3** |  |  |
| Oishi K | 1 | 1 | 1 | 1 | 0 | 0 | 1 | 0 | 0 | Fair | No follow up data |
| Takahashi S | 0 | 1 | 1 | 1 | 1 | 0 | 1 | 0 | 0 | Fair | 97.5% of participants male, no follow up data |
| Nishimura K | 1 | 1 | 1 | 1 | 0 | 0 | 1 | 0 | 0 | Fair | No follow up data |
| Kagiali S | 1 | 1 | 1 | 1 | 1 | 0 | 1 | 0 | 0 | Fair | No follow up data |
| Dias LS | 1 | 1 | 1 | 1 | 1 | 1 | 1 | 0 | 0 | Good | Patients exclusively enrolled from public centre providing free medicines, no follow up data |
| Gale NS | 1 | 1 | 1 | 1 | 1 | 1 | 1 | 0 | 0 | Good | No follow up data |
| Medina-Mirapeix F | 1 | 1 | 1 | 1 | 1 | 0 | 1 | 0 | 0 | Fair | No follow up data |
| Naval E | 1 | 1 | 1 | 1 | 1 | 0 | 1 | 0 | 0 | Fair | No follow up data |
| Hirai K | 1 | 1 | 1 | 1 | 0 | 0 | 1 | 0 | 0 | Fair | No follow up data |
| Chen PJ | 0 | 1 | 1 | 1 | 0 | 0 | 1 | 0 | 0 | Fair | 100% of participants male, no follow up data |
| Mustafaoğlu BT | 1 | 1 | 1 | 1 | 0 | 0 | 1 | 0 | 0 | Fair | No follow up data |
| ter Beek L | 1 | 1 | 1 | 1 | 0 | 0 | 1 | 0 | 0 | Fair | No follow up data |
| Finamore P | 1 | 1 | 1 | 1 | 1 | 0 | 1 | 0 | 0 | Fair | Pulmonary rehabilitation, 6-month follow up |
| Gephine S | 1 | 1 | 1 | 1 | 1 | 0 | 1 | 0 | 0 | Fair | No follow up data |
| Yee N | 1 | 1 | 1 | 1 | 1 | 1 | 1 | 1 | 1 | Good | 2-year follow up data |
| Scarlata S | 1 | 1 | 1 | 1 | 1 | 1 | 1 | 1 | 1 | Good | 4-year follow up data |
| Luo J | 1 | 1 | 1 | 1 | 1 | 1 | 1 | 1 | 1 | Good | 1-year follow up data |
| Kennedy CC | 1 | 1 | 1 | 1 | 1 | 1 | 1 | 1 | 1 | Good | 2-year follow up data |
| Warwick M | 1 | 1 | 1 | 1 | 1 | 0 | 1 | 1 | 1 | Good | Hospital mortality for AECOPD in ICU, 30-day readmission |
| Gu J-J | 1 | 1 | 1 | 1 | 1 | 0 | 1 | 0 | 0 | Fair | Hospital mortality for AECOPD |
| Alqahtani JS | 1 | 1 | 1 | 1 | 1 | 1 | 1 | 1 | 1 | Good | 30-day and 90-day follow up data after AECOPD |
| Witt LJ 2021 | 1 | 1 | 1 | 1 | 1 | 1 | 1 | 1 | 1 | Good | 30-day follow up data after AECOPD |
| Castellana F | 1 | 1 | 1 | 1 | 0 | 0 | 1 | 0 | 0 | Fair | No follow up data in COPD group |
| Lee SY | 1 | 1 | 1 | 1 | 1 | 1 | 1 | 1 | 1 | Good | 8-year follow up data |
| Ierodiakonou D | 1 | 1 | 1 | 1 | 1 | 1 | 1 | 0 | 0 | Good | No follow up data |

Domain scored^&^: Good (3+); fair (2); poor (0-1)

Domain scored*: Good (2-3); fair (1); poor (0)

**For a study to be classed as good quality, it had to score ‘good’ for every domain, two domains were deemed as fair quality and one or no domains as poor quality

**SI-Table 1B. Newcastle-Ottawa Score domain description.**

| Domain |  | Domain description |
| --- | --- | --- |
| Selection | 1 | Representativeness of the exposed cohort of the overall community |
|  | 2 | Selection of the non exposed cohort from the same community as the exposed cohort |
|  | 3 | Ascertainment of exposure; reliable method of data collection used. |
|  | 4 | Demonstration that outcome of interest was not present at start of study |
| Comparability | 1 | Comparability of groups |
|  | 2 | Adjusted in the analysis |
| Outcome | 1 | Assessment of outcome; reliable method of data collection used. |
|  | 2 | Was follow-up long enough for outcomes to occur? |
|  | 3 | Adequacy of follow up of cohorts; were all subjects accounted for? |

**SI-Table 1C. The Included Studies on COPD for Frailty Prevalence Estimation.**

|  |  |  |  |  | |  |  |  |  |
| --- | --- | --- | --- | --- | --- | --- | --- | --- | --- |
| **Author** | **Year** | **No. patients** | **Age** | **Frailty scale** | **Frailty Prevalence in COPD (%)** | | **Frailty Status**  **(non-frail, pre-frail, frail) in COPD (%)** | **Key findings** |  |
| **Studies on COPD and frailty** | | | | | | | | |  |
| Oishi K et al. | 2020 | 128 | ≥40 yrs    73 (69-78) median | Kihon checklist | 48 (37.5%) | | 23.4% - 39% - 37.5% | Positive association between limited activities of daily living and prevalence of frailty. |  |
| Takahashi S et al. | 2021 | 40 | No age cut off  70.6 ± 8.2 | Kihon Checklist | 20/40 (50%) | | 22.5% - 27.5% - 50% | Poor quality of life associated with frailty and depressive symptoms (p <0.001).  Lower hippocampal volume in COPD with frailty (p <0.05). |  |
| Nishimura K et al. | 2021 | 89 | ≥50 yrs  78 (74-82) | Kihon Checklist | 29/89 (32.6%) | | 41.6% - 25.8% - 32.6% | Correlation between pain score and frailty score and fatigue score (RS = 0.293 and 0.233, respectively). |  |
| Kagiali S et al. | 2021 | 48 | ≥55 yrs  67.3 ± 5.1  Frail COPD patients  65.1 ± 4.6  Non-frail COPD patients | Fried frailty phenotype | 20/48 (41.6%) | | 58.4% - NA - 41.6% | The performance of activities of daily living was impaired in the frail group (p <0.05). |  |
| Dias LS et al. | 2020 | 153 | ≥40 yrs  68.8 (60.5-80.5) | FRAIL scale | 77/153 (50.3%) | | 14.4% - 35.3% - 50.3% | Increased chance of frailty in COPD GOLD* group B (p =0.04) and GOLD group D (p =0.02).  No association between frailty and exacerbations in the previous year. |  |
| Gale NS et al. | 2018 | 670 | No age cut off  66.1 ± 7.6 | Frailty Index-CGA | 143/520 COPD (27.5%) | | 72.5% - NA - 27.5% | Number of exacerbations in the previous year was predictor of frailty (β =0.11). |  |
| Medina-Mirapeix F et al. | 2018 | 137 | 40-80 yrs  66.9 ± 8.3 | Fried Frailty Phenotype | 12/137 (8.8%) | | 17.5% - 73.7% - 8.8% | No association between frailty and exacerbations in the previous year. |  |
| Naval E et al. | 2021 | 127 | ≥40 yrs  66.5 ± 7.9 | Fried Frailty Phenotype | 31/127 (24.4%) | | 25.2% - 50.4% - 24.4% | Number of exacerbations in the previous year and anxiety and depressive symptoms were associated with frailty (p <0.001). |  |
| Hirai K et al. | 2019 | 201 | ≥65 yrs  76 (70-81) median | Kihon checklist | 76/201 (38%) | | 26% - 36% - 38% | Correlation of frailty with anxiety and depressive symptoms (p <0.001). |  |
| Chen PJ et al. | 2018 | 125 | No age cut off  76.3 ± 10.2 | Canadian Study of Health and Aging Clinical Frailty Scale with score modified for the Taiwan study | - | | 86% in dyspnea group  26.7% in non-dyspnea group | COPD patients divided into dyspnea group and non-dyspnea group according to the modified Medical Research Council dyspnea scale. Higher prevalence of frailty in COPD patients with dyspnea compared to COPD patients without dyspnea.  Number of exacerbations in the last year associated with frailty in the dyspnea group (p <0.001). |  |
| Mustafaoğlu BT et al. | 2020 | 61 | Range 65-84 | Tilburg Frailty Indicator | 39/61 (64%) | | 36.1% - NA - 64% | Positive association between frailty score and number of comorbidities (p = 0.007). |  |
| ter Beek L et al. | 2020 | 57 | ≥40 yrs  61.2 ± 8.7 | Fried Frailty Phenotype | 16/57 (28%) | | 8.8% - 63.2% - 28% | Coexistence of frailty and malnutrition in 40%. |  |
| Finamore P et al. | 2021 | 53 | No age cut off  73 ± 8 | PRISMA-7 questionnaire | 38/53 (72%) | | 28% - NA - 72% | COPD outpatients in GOLD* 1-3 grades enrolled in pulmonary rehabilitation programme (excluding patients with very severe airflow limitation).  Frailty associated with distance at 6MWT^§^ during (p =0.01) and after (p =0.02) pulmonary rehabilitation with higher potential to benefit in frail patients. |  |
| Gephine S et al. | 2021 | 44 | ≥40 yrs  66 ± 8 | Fried Frailty Phenotype | 19 (43%) | | 57% - NA - 43% | COPD patients with chronic respiratory failure and living with frailty had higher fatigue, anxiety and depressive symptoms, and greater use of nutritional supplements (p <0.05).  No association between frailty and exacerbations in the previous year. |  |
| **Cohort studies on COPD outpatients and frailty** | | | | | | | | |  |
| Yee N et al. | 2020 | 280 | ≥40 yrs  68 mean | Fried Frailty Phenotype | 64 (23%) | | 14% - 63% - 23% | COPD patients living with frailty had increased risk of non-COPD hospitalisations. |  |
| Scarlata S et al. | 2021 | 150 | ≥40 yrs  73 ± 8 | Frailty Index | 71 (47.4%) | | NA - 52.6% - 47.4% | Frailty score higher in frequent exacerbators (p <0.01). |  |
| Luo J et al. | 2021 | 309 | ≥65 yrs  86 (80-90) median | Fried Frailty Phenotype | 154/309 (49.8%) | | 50.2% - NA - 49.8% | COPD living with frailty had increased risk of acute exacerbations (IRR =1.75, 95%CI 1.09-2.82), all-cause hospitalisations (IRR =1.39, 95%CI 1.04-2.25), and all-cause mortality (HR =2.54, 95%CI 1.01-6.36). |  |
| Kennedy CC et al. | 2019 | 902 | 67 (63-70) median | Fried Frailty Phenotype | 57/886 (6.4%) | | 31.8% - 61.7% - 6.4% | Frailty associated with increased rate of hospitalisations (aHR =1.6, 95%CI 1.1-2.5) and mortality (aHR =1.4, 95%CI 0.97-2.0). |  |
| **Cohort studies on patients admitted with acute exacerbation of COPD** | | | | | | | | |  |
| Warwick M et al. | 2021 | 390 | ≥18 yrs  63.5 (58-71) | Clinical Frailty Scale | 147/390 (37.8%) | | NA - NA - 37.8% | Pre-admission frailty as a predictor of hospital mortality (OR =4.12, 95%CI 2.26-6.95). |  |
| Gu J-J et al. | 2021 | 154 | ≥60 yrs  79.7 ± 8.3 | Frailty Index-Lab | FI-Lab ≥ 0.4 44% | | FI-Lab values of 0.2-0.39 in 56%; values of ≥ 0.4 in 44%. | Frailty associated with hospital mortality: high score of frailty (FI-Lab values of ≥0.4) in 13% of survivors and 75.3% of non-survivors. |  |
| Alqahtani JS et al. | 2021 | 82 | No age cut off  71 ± 10.4 | Reported Edmonton Frail Scale | Frailty score at baseline, median 10 (9-12.2) | | No data on frailty prevalence in COPD patients assessed | Frailty was the best predictor of 30-day readmission (OR =1.72, 95%CI 1.32-2.29).  Number of exacerbations within last 12 months associated with frailty in patients readmitted within 30 days (p =0.01). |  |
| Witt LJ et al. | 2021 | 70 | ≥18 yrs  63.5 (58-71) | Fried frailty phenotype | 55/70 completed Fried Frailty measures, prevalence 67% | | 33% - NA - 67% | All patients readmitted within 30 days after admission for acute exacerbation were frail (p =0.04). |  |
| **Studies on community-dwelling adults and frailty** | | | | | | | | |  |
| Castellana F et al. | 2021 | 1929  (343 COPD) | ≥65 yrs  73.5 ± 6.2 | Fried frailty phenotype | COPD subjects with frailty 19.9% | | 15.4% - 19.6% - 19.9% | Direct relationship between COPD and frailty (p = 0.02). |  |
| Lee SY et al. | 2021 | 4627  (1162 COPD) | ≥55 yrs  66.4 ± 7.7 | Fried frailty phenotype | 6.8% in COPD group | | 44.4% - 48.8% - 6.8% | Frailty prevalence higher in COPD (6.8%) *vs* non-COPD (3.2%): OR =1.86, 95%CI 1.35-2.56 in base model adjustment for age, sex, ethnicity.  Frailty and pre-frailty associated with increased risk of mortality. |  |
| Ierodiakonou D et al. | 2019 | 257 | No age cut off  65 ± 12.3 | FiND Questionnaire | 177/253 (70%) | | 17.8% non-frail  6% frailty without disability  64% frailty with disability  12.2% only disability | COPD patients assessed in primary care setting.  Frailty associated with number of exacerbations in the last 12 months (p =0.033). |  |
|  | | | | | |  |  |  |  |
|  |  |  |  |

*www.goldcopd.org

^§^6MWT = 6-minute walk test

**SI-Table 1D. The frailty assessment tools used in the included studies.**

| **References/Year** | **Frailty Scale** | **Items measured** | **Scoring** | **Administration** |
| --- | --- | --- | --- | --- |
| Fried et al. 2001 | **Fried Frailty phenotype**  (Physical Frailty Phenotype) | 5 domains:  Slowness  Physical activity  Weight loss  Exhaustion  Weakness | Score range: 0 to 5.  Frail = ≥3 criteria  Pre-frail = 1-2 criteria  Non-frail = 0 | Physician and self-reported |
| Mitnitski et al. 2001; Rockwood et al. 2007 | **Frailty Index**  (Deficit Accumulation Index) | Scales vary in content and number of items, generally 30-70.  Multiple domains including laboratory findings, physical function disabilities, diseases, symptoms, sensory difficulties, cognition difficulties | Number of deficits present and divided by the number of deficits considered. Higher proportion = higher level of frailty. | Physician |
| Rockwood et al. 2011 | **Frailty Index-Comprehensive Geriatric Assessment**  (Modified for community-dwelling individuals) | Motivation, self-rated health, cognition, emotional, sleep, communication, strength, mobility, balance, elimination, nutrition, ADLs, IADLs, social engagement, medical history | Dividing the total number of CGA deficits by the maximum score of 61. | Physician |
| Howlett et al. 2014; Rockwood et al. 2015 | **Frailty Index-Lab**  Frailty Index based on routine laboratory tests | 23 domains:  21 blood tests (albumin, aspartate aminotransferase, calcium, creatinine, folate, folate red blood cells, fasting glucose, hemoglobin, mean corpuscular volume, alkaline phosphatase, inorganic phosphorus, potassium, total protein, sodium, TSH, Thyroxine, Venereal Disease Research Laboratory, free T4, urea, vitamin B12, white blood cells) PLUS arterial blood pressure values | Number of deficits present and divided by the number of deficits measured. | Physician |
| Abellan van Kan et al. 2008; Morley et al. 2012 | **FRAIL Scale** | 5 domains:  Fatigue  Resistance  Ambulation  Illnesses  Loss of weight | Score range: 0 to 5.  Non-frail = 0 deficits  Intermediate frail = 1 or 2 deficits  Frail = 3 or more deficits | Self-reported |
| Rolfson et al. 2006 | **Reported Edmonton Frail Scale (REFS)** | 10 domains:  Cognition  Functional performance (balance and mobility)  Self-reported items: general health, functional independence, social support, medication use, nutrition, mood, continence | Each item is scored, and then all scores are summed out of 18 points possible.  Non frail = 0-7  Mild frail = 8-9  Moderate frail = 10-11  Severe frail = 12-18 | Physician and self-reported |
| Rockwood et al. 2005 | **Clinical Frailty Scale (CFS)** | 9 categories:   1. Very fit (robust, active) 2. Well (no active diseases but less fit than “very fit”) 3. Managing well (medical problems are well controlled; people not regularly active) 4. Vulnerable (not dependent but symptoms limit activities) 5. Mildly frail (limited dependence in high order IADLs) 6. Moderately frail (help needed with outside activities, with keeping house, with bathing) 7. Severely frail (completely dependent but stable) 8. Very severely frail (completely dependent, approaching the end of life) 9. Terminally ill (approaching the end of life, with life expectancy < 6 months) | A physician assigns score of 1 to 7 based on clinical judgement. Physicians making the initial assessment given access to diagnoses and review of medical records. | Physician |
| Chan et al. 2010; Searle et al. 2008; Rockwood et al. 2010 | **Canadian Study of Health and Aging Clinical Frailty Scale (CSHA-CFS)** | ADLs (eating, dressing, transferring, toileting, bathing), IADLs (shopping, taking medications, using telephone, financing, transportation, preparing meals, doing light house works, doing laundry) | Based on combination of answers Yes or No, a category of CFS is assigned. | Physician by phone interview |
| Cesari et al. 2014 | **Frail Non-Disabled (FiND) questionnaire** | 5 questions:  Disability: A difficulty at walking; B difficulty at climbing up  Frailty: C weight loss; D limited activities; E level of physical activity | Disabled = A+B ≥ 1  Frail = A+B = 0 and C+D+E = ≥ 1  Non-frail = A+B+C+D+E = 0 | Physician |
| Tomata et al. 2011 | **Kihon Checklist** | 25-item questionnaire including 7 domains: instrumental activity of daily living, social activity of daily living, physical strength, nutritional status, oral function, cognitive status, depression risk | Non-frail = 0-3  Pre-frail = 4-7  Frail = ≥ 8 | Physician |
| Gobbens et al. 2010 | **Tilburg Frailty Indicator** | 15 domains including physical, psychological and social domains | Score 0-15 | Physician |
| Turner G et al. 2014; Raiche M et al. 2008 | **PRISMA-7 questionnaire** | 7 yes/no questions about:  1) Age; 2) Gender; 3) Health problems that limit activities; 4) Help needed from someone regularly; 5) Health problems that require staying at home; 6) Having someone to count on if needed; and 7) Regular use of an assistive device for walking. | Answering yes to three or more of the seven questions = potential disabilities/frailty | Self-reported |
| Cacciatore F et al. 2005; Lachs MS et al. 1990 | **Frailty staging system** | 7 domains: disability, mobility, cognitive function, visual function, hearing function, incontinence, social support | Score 0 = function preserved; score 1 = function lost in each domain. Subjects who are frail in one or more domains are considered frail; subjects who are not frail in any domain are considered not frail. | Physician |

| **SI-Table 1E. Studies included in Marengoni *et al.* (2018) and considered in the analysis of frailty prevalence estimate, mortality and readmission.** | | | | | | | | | |
| --- | --- | --- | --- | --- | --- | --- | --- | --- | --- |
| **Author** | **Year** | **Country** | **No. of patients and population** | **Sex** | **Age** | **COPD diagnosis/**  **Definition criteria** | **Frailty measure** | **Frailty prevalence in COPD** | **Pre-frailty prevalence in COPD** |
| Galizia G et al. | 2011 | Italy | 1288, community-dwelling older adults | 43% M  57% F | ≥65 yrs  74.2 ± 6.3 | GOLD guidelines | Frailty staging system (7 domains) | **48.9%** | - |
| Lahousse L et al. | 2016 | The Netherlands | 2142, community-dwelling older adults | 45.7%  979 M  54.3%  1163 F | 74.7 ± 5.6  range  65-97 | Post-BD FEV_1_/FVC < 0.7 and absence of asthma | Fried frailty phenotype | **10.2%** | 9.6% |
| Maddocks M et al. | 2016 | UK | 816, outpatients referred to pulmonary rehabilitation | 59.3%  484 M  40.7%  332 F | ≥35 yrs  69.8 ± 9.7 | GOLD guidelines | Fried frailty phenotype | **25.6%** | 64.3% |
| Bernabeu-Mora R et al. | 2017 | Spain | 103, COPD patients hospitalized for AECOPD | 93.2%  96 M  6.8%  7 F | 71 ± 9.1 | GOLD guidelines | Reported Edmonton Frailty Scale | **56.3%** | - |
| Limpawattana P et al. | 2017 | Thailand | 121, COPD outpatients | 92.6%  112 M  7.4%  9 F | ≥18 yrs  73.5 ± 8.9 | GOLD guidelines | FRAIL scale | **6.6%** | 41.3% |
| Kusunose M et al. | 2017 | Japan | 79, COPD outpatients | - | ≥50 yrs  74.8 ± 6.3  range 60-88 | GOLD guidelines | Kihon checklist | **21.5%** | 30.4% |

**SI-Figure 3. Subgroup sensitivity analysis of frailty on all-cause long-term mortality in COPD across six studies.**


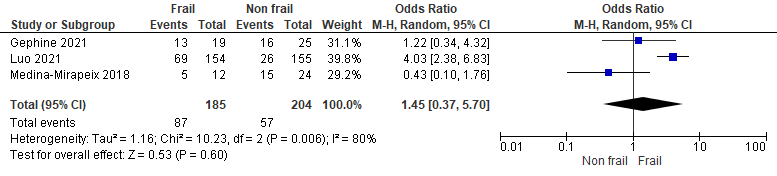


**SI-Figure 4. A meta-analysis of frailty and past exacerbations in COPD across three studies.**

**SI-Table 1F. Search strategy.**

Embase Classic+Embase <1947 to 2022 March 24>

Search strategy:

---------------------------------------------------------------------------------------------------------------------------------------------

1 (frailty and COPD).mp. [mp=title, abstract, heading word, drug trade name, original title, device manufacturer, drug manufacturer, device trade name, keyword heading word, floating subheading word, candidate term word] 469

2 (frailty and emphysema).mp. [mp=title, abstract, heading word, drug trade name, original title, device manufacturer, drug manufacturer, device trade name, keyword heading word, floating subheading word, candidate term word] 66

3 (frailty and pulmonary disease).mp. [mp=title, abstract, heading word, drug trade name, original title, device manufacturer, drug manufacturer, device trade name, keyword heading word, floating subheading word, candidate term word] 502

4 (frailty and chronic obstructive bronchitis).mp. [mp=title, abstract, heading word, drug trade name, original title, device manufacturer, drug manufacturer, device trade name, keyword heading word, floating subheading word, candidate term word] 1

**SI-Table 1G. PRISMA 2020 Checklist.**


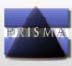
**PRISMA 2020 Checklist**

| **Section and Topic** | **Item #** | **Checklist item** | **Location where item is reported** |
| --- | --- | --- | --- |
| **TITLE** | | |  |
| Title | 1 | Identify the report as a systematic review. |  |
| **ABSTRACT** | | |  |
| Abstract | 2 | See the PRISMA 2020 for Abstracts checklist. |  |
| **INTRODUCTION** | | |  |
| Rationale | 3 | Describe the rationale for the review in the context of existing knowledge. |  |
| Objectives | 4 | Provide an explicit statement of the objective(s) or question(s) the review addresses. |  |
| **METHODS** | | |  |
| Eligibility criteria | 5 | Specify the inclusion and exclusion criteria for the review and how studies were grouped for the syntheses. |  |
| Information sources | 6 | Specify all databases, registers, websites, organisations, reference lists and other sources searched or consulted to identify studies. Specify the date when each source was last searched or consulted. |  |
| Search strategy | 7 | Present the full search strategies for all databases, registers and websites, including any filters and limits used. |  |
| Selection process | 8 | Specify the methods used to decide whether a study met the inclusion criteria of the review, including how many reviewers screened each record and each report retrieved, whether they worked independently, and if applicable, details of automation tools used in the process. |  |
| Data collection process | 9 | Specify the methods used to collect data from reports, including how many reviewers collected data from each report, whether they worked independently, any processes for obtaining or confirming data from study investigators, and if applicable, details of automation tools used in the process. |  |
| Data items | 10a | List and define all outcomes for which data were sought. Specify whether all results that were compatible with each outcome domain in each study were sought (e.g. for all measures, time points, analyses), and if not, the methods used to decide which results to collect. |  |
|  | 10b | List and define all other variables for which data were sought (e.g. participant and intervention characteristics, funding sources). Describe any assumptions made about any missing or unclear information. |  |
| Study risk of bias assessment | 11 | Specify the methods used to assess risk of bias in the included studies, including details of the tool(s) used, how many reviewers assessed each study and whether they worked independently, and if applicable, details of automation tools used in the process. |  |
| Effect measures | 12 | Specify for each outcome the effect measure(s) (e.g. risk ratio, mean difference) used in the synthesis or presentation of results. |  |
| Synthesis methods | 13a | Describe the processes used to decide which studies were eligible for each synthesis (e.g. tabulating the study intervention characteristics and comparing against the planned groups for each synthesis (item #5)). |  |
|  | 13b | Describe any methods required to prepare the data for presentation or synthesis, such as handling of missing summary statistics, or data conversions. |  |
|  | 13c | Describe any methods used to tabulate or visually display results of individual studies and syntheses. |  |
|  | 13d | Describe any methods used to synthesize results and provide a rationale for the choice(s). If meta-analysis was performed, describe the model(s), method(s) to identify the presence and extent of statistical heterogeneity, and software package(s) used. |  |
|  | 13e | Describe any methods used to explore possible causes of heterogeneity among study results (e.g. subgroup analysis, meta-regression). |  |
|  | 13f | Describe any sensitivity analyses conducted to assess robustness of the synthesized results. |  |
| Reporting bias assessment | 14 | Describe any methods used to assess risk of bias due to missing results in a synthesis (arising from reporting biases). |  |
| Certainty assessment | 15 | Describe any methods used to assess certainty (or confidence) in the body of evidence for an outcome. |  |
| **RESULTS** | | |  |
| Study selection | 16a | Describe the results of the search and selection process, from the number of records identified in the search to the number of studies included in the review, ideally using a flow diagram. |  |
|  | 16b | Cite studies that might appear to meet the inclusion criteria, but which were excluded, and explain why they were excluded. |  |
| Study characteristics | 17 | Cite each included study and present its characteristics. |  |
| Risk of bias in studies | 18 | Present assessments of risk of bias for each included study. |  |
| Results of individual studies | 19 | For all outcomes, present, for each study: (a) summary statistics for each group (where appropriate) and (b) an effect estimate and its precision (e.g. confidence/credible interval), ideally using structured tables or plots. |  |
| Results of syntheses | 20a | For each synthesis, briefly summarise the characteristics and risk of bias among contributing studies. |  |
|  | 20b | Present results of all statistical syntheses conducted. If meta-analysis was done, present for each the summary estimate and its precision (e.g. confidence/credible interval) and measures of statistical heterogeneity. If comparing groups, describe the direction of the effect. |  |
|  | 20c | Present results of all investigations of possible causes of heterogeneity among study results. |  |
|  | 20d | Present results of all sensitivity analyses conducted to assess the robustness of the synthesized results. |  |
| Reporting biases | 21 | Present assessments of risk of bias due to missing results (arising from reporting biases) for each synthesis assessed. |  |
| Certainty of evidence | 22 | Present assessments of certainty (or confidence) in the body of evidence for each outcome assessed. |  |
| **DISCUSSION** | | |  |
| Discussion | 23a | Provide a general interpretation of the results in the context of other evidence. |  |
|  | 23b | Discuss any limitations of the evidence included in the review. |  |
|  | 23c | Discuss any limitations of the review processes used. |  |
|  | 23d | Discuss implications of the results for practice, policy, and future research. |  |
| **OTHER INFORMATION** | | |  |
| Registration and protocol | 24a | Provide registration information for the review, including register name and registration number, or state that the review was not registered. |  |
|  | 24b | Indicate where the review protocol can be accessed, or state that a protocol was not prepared. |  |
|  | 24c | Describe and explain any amendments to information provided at registration or in the protocol. |  |
| Support | 25 | Describe sources of financial or non-financial support for the review, and the role of the funders or sponsors in the review. |  |
| Competing interests | 26 | Declare any competing interests of review authors. |  |
| Availability of data, code and other materials | 27 | Report which of the following are publicly available and where they can be found: template data collection forms; data extracted from included studies; data used for all analyses; analytic code; any other materials used in the review. |  |

*From:*  Page MJ, McKenzie JE, Bossuyt PM, Boutron I, Hoffmann TC, Mulrow CD, et al. The PRISMA 2020 statement: an updated guideline for reporting systematic reviews. BMJ 2021;372:n71. doi: 10.1136/bmj.n71

For more information, visit: <http://www.prisma-statement.org/>
